# The impact of the recent AAP changes in palivizumab authorization on RSV-induced bronchiolitis severity and incidence

**DOI:** 10.1186/s13052-017-0390-8

**Published:** 2017-08-14

**Authors:** Antonino Capizzi, Michela Silvestri, Andrea Orsi, Renato Cutrera, Giovanni A. Rossi, Oliviero Sacco

**Affiliations:** 1 Department of Pediatrics, Pulmonology and Allergy Unit and Cystic Fibrosis Center, Istituto Giannina Gaslini, Genoa, Italy; 2 Department of Health Sciences, Hygiene Unit, IRCCS University Hospital San Martino Polyclinic, Genoa, Italy; 3 Pediatric Pulmonology and Sleep & Long Term Ventilation Unit, Pediatric Hospital “Bambino Gesù”, Rome, Italy

**Keywords:** Palivizumab, Respiratory syncytial virus, Prophylaxis, Preterm

## Abstract

Following the most recent modification by the American Academy of Pediatrics, based on American studies on RSV epidemiology, the Italian Drug Agency (AIFA) decided to limit the total financial coverage of the palivizumab prescription by the National Health Service only to the < 29 wGA group and age ≤ 12 months at the beginning of the RSV epidemic season. However, the vulnerability of otherwise healthy premature infants ≥ 29 wGA has been demonstrated in Italian analyses. We retrospectively reviewed records from children ≤ 1 years of age admitted for RSV-induced ALRI at the Gaslini Hospital, over three consecutive RSV epidemic seasons (RES) (2014–2017). We found that the prescription limitation on RSV immunoprophylaxis in preterms was associated in the 2016–2017 RES with: a) a high proportion of admission for the < 36 wGA infants, the great majority born at 33- < 36 wGA and a chronological age of < 6 months; b) a high proportion of preterms treated with high flow nasal cannula ventilation. These results strongly point to a need to reevaluate the role of palivizumab prophylaxis in the >= 29 wGA subpopulation when specific risk factors are present.

## Dear Editor,

**Fig. 1:**
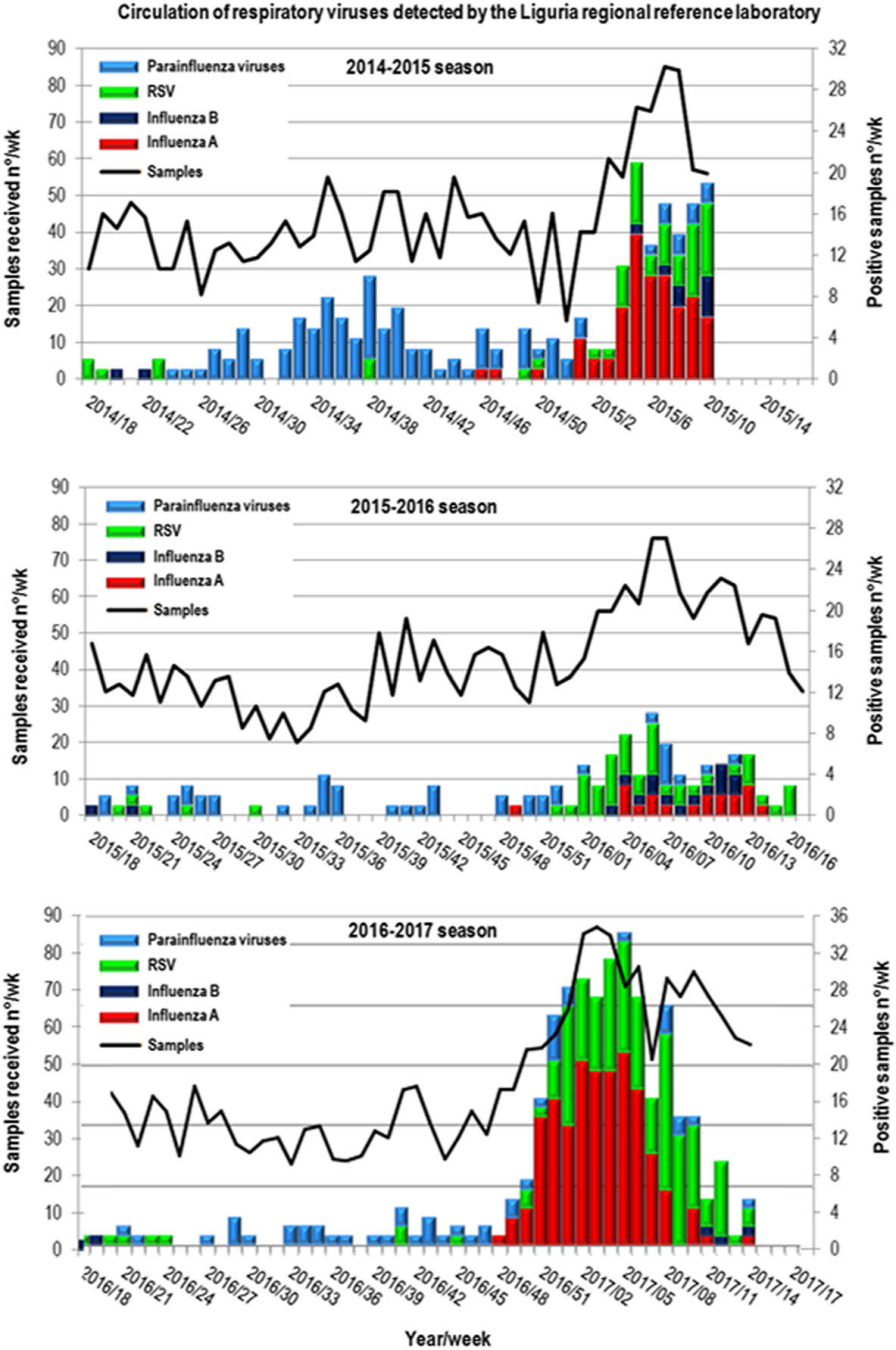
Circulation of respiratory viruses detected by the Liguria regional reference laboratory in the three RSV epidemic seasons (2014–2015, 2015–2016 and 2016–2017): data related to RSV are reported in *green* columns

Respiratory syncytial virus (RSV) is the single most important cause of acute lower respiratory tract infection (ALRI) in infants, associated with significant morbidity and, sometimes, mortality in industrialized nations [1]. In clinical trials, palivizumab reduced RSV hospitalization rates for premature infants [1]. The current Italian Guidelines recommend palivizumab prophylaxis for infants of 29–35 weeks gestational age (wGA) and a chronological age ≤ 6 months at the beginning of the epidemic season, in presence of risk conditions predisposing the infant to severe infections and/or need for hospitalization [2]. These include attendance of the child in a community setting and/or presence of one or more cohabitees younger than 5 years [3]. However, following the most recent modification by the American Academy of Pediatrics based on American studies on RSV epidemiology [4], in September 2016, the Italian Drug Agency (AIFA) decided the total financial coverage of the palivizumab prescription to the healthy preterms by the National Health Service, should be limited to the < 29 wGA group and age ≤ 12 months at the beginning of the RSV epidemic season (RES) [5]. However, the vulnerability of otherwise healthy premature infants, and most notably in the < 32 wGA category was demonstrated by an Italian retrospective analysis on RSV-associated hospitalization during the RSV epidemic season, over a 4 year period, when the use of prophylaxis palivizumab was not widespread in premature infants [6]. Moreover, a recent study on total costs for hospitalizations and emergency room and/or outpatient visits in infants in the first 3 years of life in Lombardy (Italy) showed that extending palivizumab prophylaxis to 29–32 wGA infants appeared to be a cost-effective strategy [7]. With this background we retrospectively reviewed records from children ≤ 1 years of age admitted, for RSV-induced ALRI at the Gaslini Hospital, over three consecutive RES (2014–2017) (Fig. 1). The possible impact on the incidence and severity of RSV bronchiolitis after the new prescription limitation was evaluated, comparing the third RES (2016–2017), with the two previous ones.

**Fig. 2:**
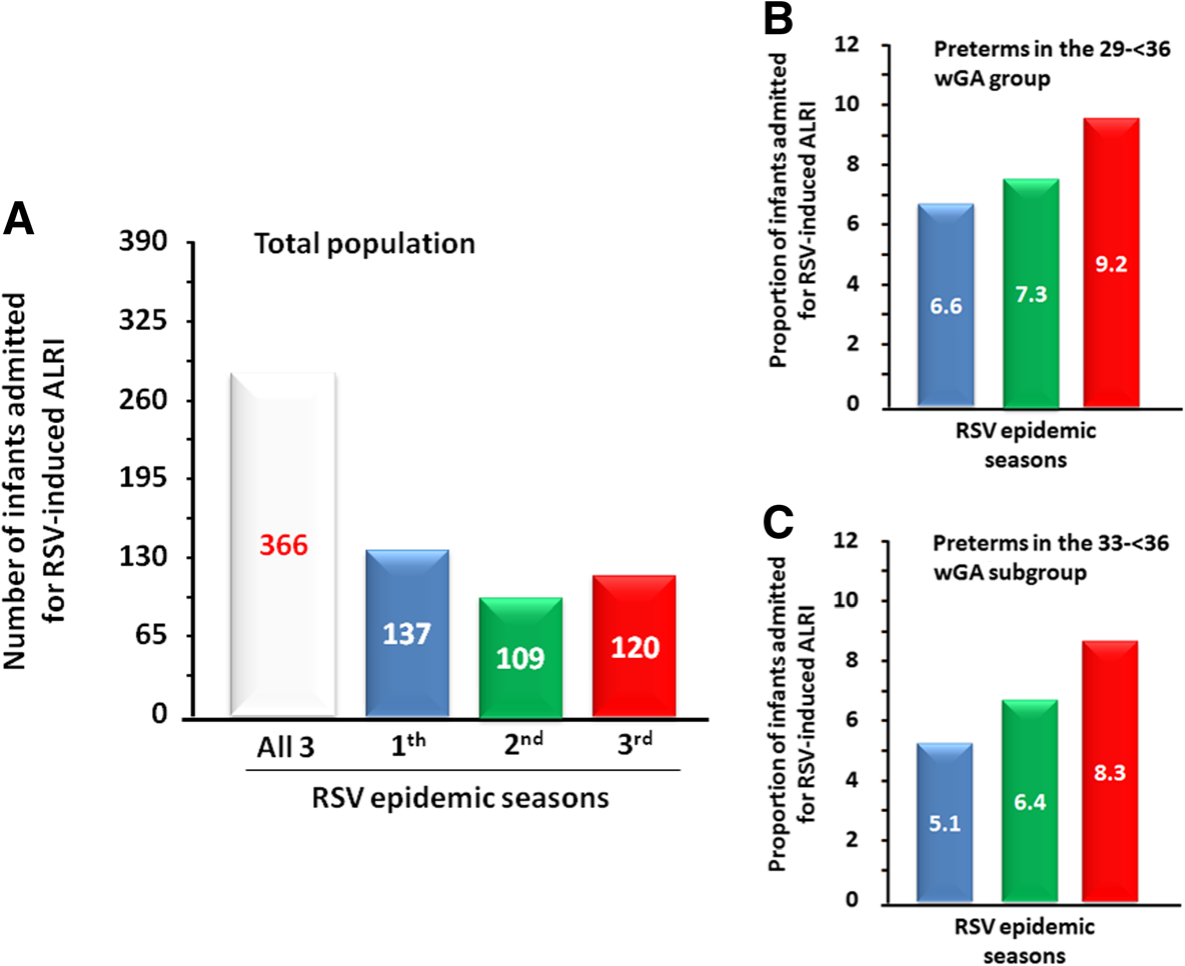
Admission for RSV infection in infants. **a** Number of infants admitted in the three RSV epidemic seasons (2014–2015, 2015–2016 and 2016–2017). **b** Proportion of preterms (29- < 36 wGA) admitted in each RSV epidemic season. **c** Proportion of preterms (33- < 36 wGA) admitted in each RSV epidemic season

**Fig. 3:**
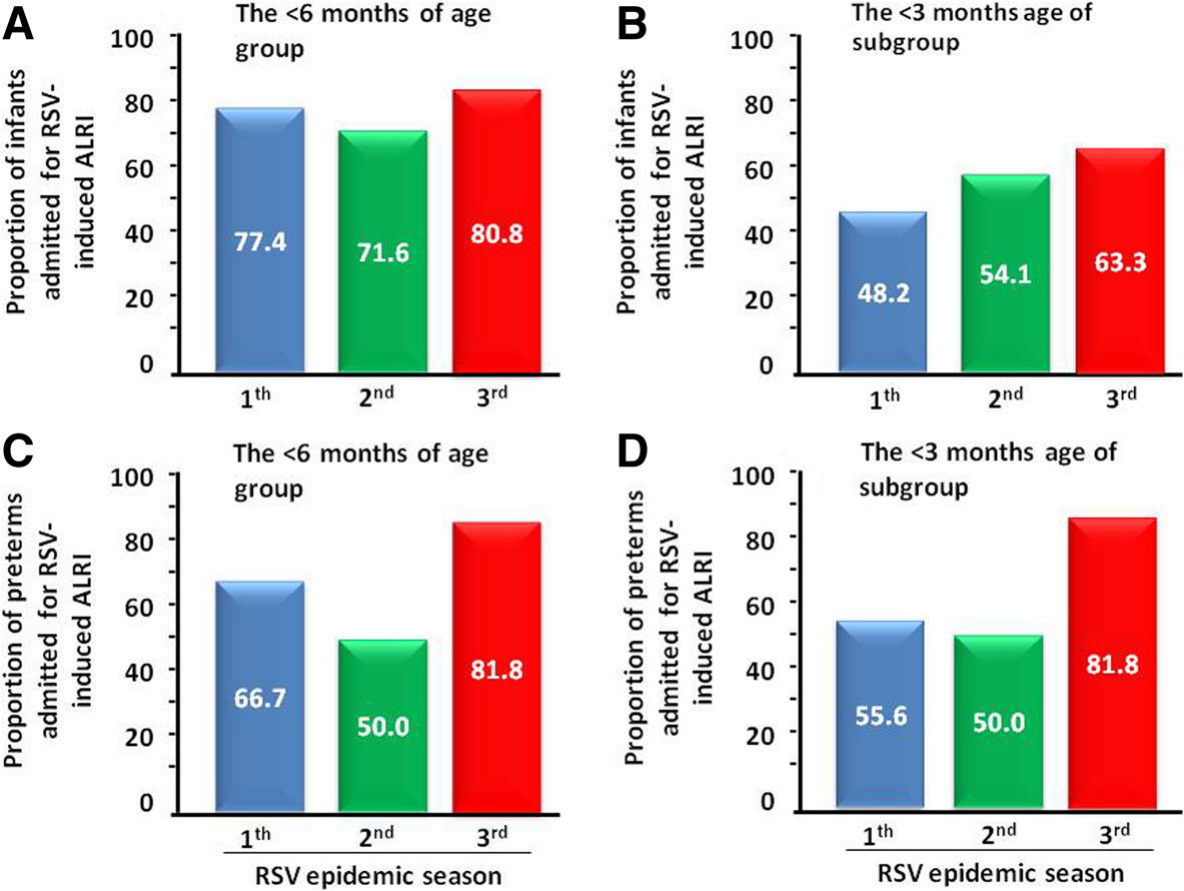
Proportion of infants (panel **a** and **b**) or of preterms (panel **c** and **d**) admitted for RSV infection in the three RSV epidemic seasons (2014–2015, 2015–2016 and 2016–2017). **a** and **c** Infants < 6 months of age; (**b** and **d**). Subgroup of infants < 3 months of age

All the infant included in the study, had a nasal swab positive for RSV. Co-infections were detected only in 4 infants (*Haemophilus influenza*) with a wGA < 34 weeks. The total number of infants admitted for RSV-induced ALRI in the three RSV epidemic seasons was 366: 137 in the 1th, 109 in the 2nd and 120 in the 3rd season (Fig. 2a). Only 7.7% of these infants were preterms (29- < 36 wGA), 6.6% in the 33- < 36 wGA subgroup and only 1.1% in the 29- < 33 wGA subgroup. Interestingly, the proportion of preterms admitted tended to increased in the three RES from 6.6%, to 7.3%, and to 9.2%, respectively, for the 29- < 36 wGA group (Fig. 2b), and from 5.1% to 6.4% and to 8.3%, respectively, for the 33- < 36 wGA subgroup (Fig. 2c). Due to the due to small sample size these increases are not statistically significant. The proportion of the very young infants admitted with a chronological age of < 6 months tended to increase in the three RSV epidemic seasons from 77.4%, 71.6% and 80.8% (Fig. 3a), and this tendency was even more evident in the < 3 months chronological age group (48.2%, to 54.1% and to 63.3%, respectively (Fig. 3b).

This trend was observed also in preterms admitted with a chronological age of < 6 months (66.7%, 50% and 81.8%, respectively; Fig. 3c) and in the < 3 month subgroup (55.6%, 50% and 81.8%, respectively (Fig. 3d).

**Fig. 4:**
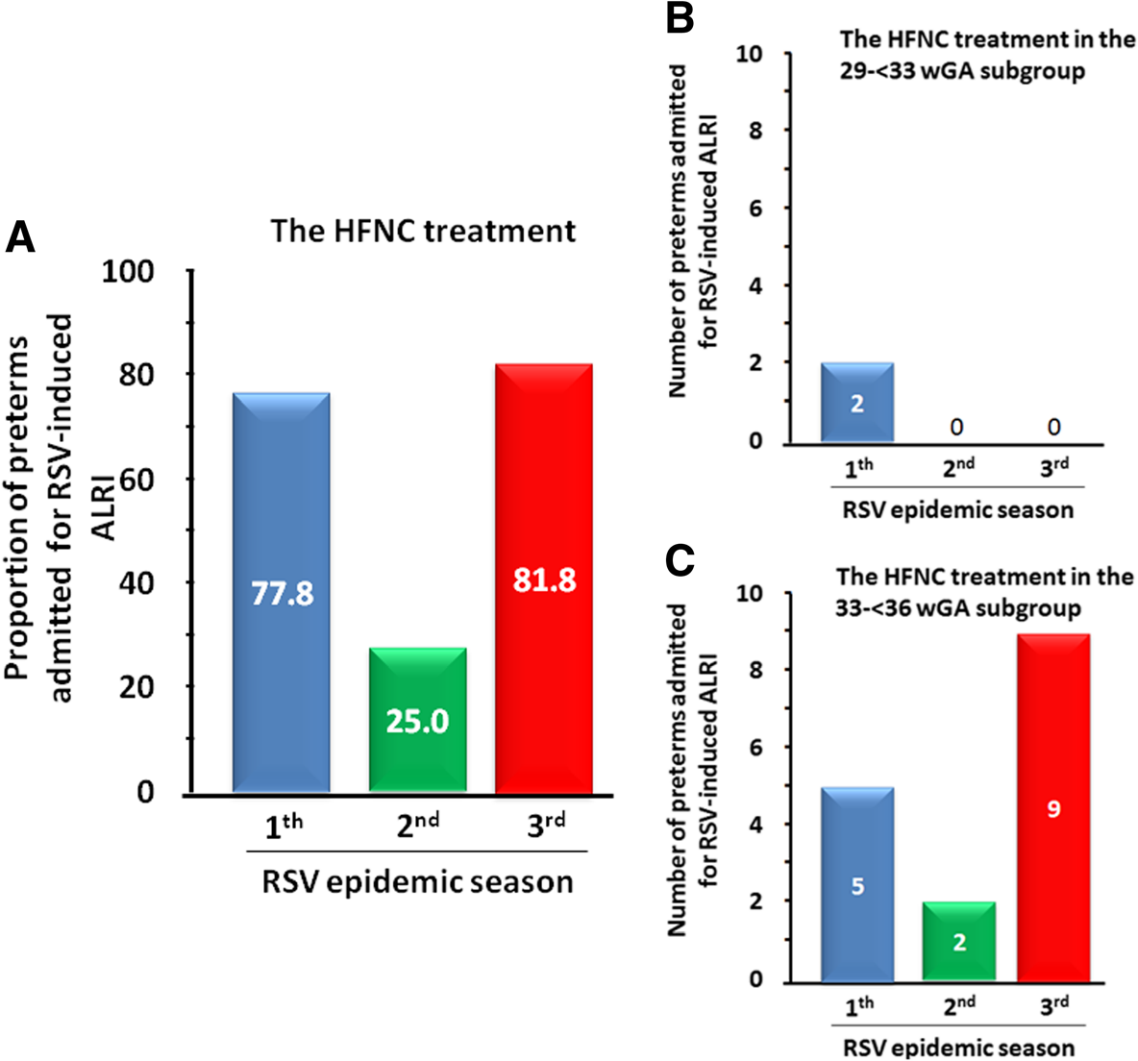
Frequency of treatment with high flow nasal cannula (HFNC) ventilation among preterms in the three RSV epidemic seasons (2014–2015, 2015–2016 and 2016–2017). **a** Whole preterm population. **b** The 29- < 33 wGA subgroup. **c** The 33- < 36 wGA subgroup

The vast majority of the 33- < 36 wGA infants had a chronological age of < 6 months at the beginning of the RSV epidemic season and had at least one risk factor [3]. In addition, 64% of the admitted < 36 wGA infants were treated with high flow nasal cannula (HFNC) ventilation: 50% of those born at 29- < 33 wGA and 66.6% of those at 33- < 36 wGA. In the three RSV epidemic seasons, the proportion of preterms treated with HFNC ventilation was 77.8%, 25.0% and 81.8%, respectively (Fig. 4a), the numbers being 5, 2, 9 in the 33- < 36 wGA subgroup (Fig. 4c).

Therefore, the prescription limitation on RSV immunoprophylaxis in preterms was associated in the 2016–2017 RSV epidemic season with: a) a high proportion of admission for the < 36 wGA infants, the great majority born at 33- < 36 wGA and with chronological age of < 6 months; b) a high proportion of preterms treated with HFNC ventilation, mostly in the at 33- < 36 wGA subgroup. The major limitation of the study is that, since the data were collected in a single tertiary level paediatric hospital, the numbers of preterm infants was very small, precluding a reliable statistical analysis. This is a common problem in these kind of reports since preterms represent only a very small proportion in the general infant population, as shown also in studies that have been cited by the American Academy of Pediatrics in the guidance for palivizumab prophylaxis [8, 9]. Data collection on national records involving other tertiary level paediatric hospitals could provide a sample size large enough to perform a reliable statistical analysis. However, our results on RSV hospitalization and HFNC treatment clearly highlight the vulnerability of young preterms, especially in this last RSV season and point to a need to reevaluate the role of palivizumab prophylaxis in the > 29 wGA subpopulation when specific risk factors are present.
